# First Occurrence of Plasmablastic Lymphoma in Adenosine Deaminase-Deficient Severe Combined Immunodeficiency Disease Patient and Review of the Literature

**DOI:** 10.3389/fimmu.2018.00113

**Published:** 2018-02-02

**Authors:** Maddalena Migliavacca, Andrea Assanelli, Maurilio Ponzoni, Roberta Pajno, Federica Barzaghi, Fabio Giglio, Francesca Ferrua, Marta Frittoli, Immacolata Brigida, Francesca Dionisio, Roberto Nicoletti, Miriam Casiraghi, Maria Grazia Roncarolo, Claudio Doglioni, Jacopo Peccatori, Fabio Ciceri, Maria Pia Cicalese, Alessandro Aiuti

**Affiliations:** ^1^San Raffaele Telethon Institute for Gene Therapy (SR-TIGET), Pediatric Immunohematology and Bone Marrow Transplantation Unit, Scientific Institute San Raffaele (IRCCS), Milan, Italy; ^2^Hematology and Bone Marrow Transplantation Unit, Scientific Institute San Raffaele (IRCCS), Milan, Italy; ^3^Pathology Unit, Scientific Institute San Raffaele (IRCCS), Milan, Italy; ^4^Vita-Salute San Raffaele University, Milan, Italy; ^5^San Raffaele Telethon Institute for Gene Therapy (SR-TIGET), Scientific Institute San Raffaele (IRCCS), Milan, Italy; ^6^Department of Radiology, Scientific Institute San Raffaele (IRCCS), Milan, Italy; ^7^Division of Stem Cell Transplantation and Regenerative Medicine, Department of Pediatrics, Institute for Stem Cell Biology and Regenerative Medicine, Stanford School of Medicine, Stanford, CA, United States

**Keywords:** adenosine deaminase-deficient severe combined immunodeficiency disease, lymphoma, plasmablastic lymphoma, primary immunodeficiency, gene therapy, gene therapy for rare diseases, review of literature

## Abstract

Adenosine deaminase-deficient severe combined immunodeficiency disease (ADA-SCID) is a primary immune deficiency characterized by mutations in the ADA gene resulting in accumulation of toxic compounds affecting multiple districts. Hematopoietic stem cell transplantation (HSCT) from a matched donor and hematopoietic stem cell gene therapy are the preferred options for definitive treatment. Enzyme replacement therapy (ERT) is used to manage the disease in the short term, while a decreased efficacy is reported in the medium-long term. To date, eight cases of lymphomas have been described in ADA-SCID patients. Here we report the first case of plasmablastic lymphoma occurring in a young adult with ADA-SCID on long-term ERT, which turned out to be Epstein–Barr virus associated. The patient previously received infusions of genetically modified T cells. A cumulative analysis of the eight published cases of lymphoma from 1992 to date, and the case here described, reveals a high mortality (89%). The most common form is diffuse large B-cell lymphoma, which predominantly occurs in extra nodal sites. Seven cases occurred in patients on ERT and two after haploidentical HSCT. The significant incidence of immunodeficiency-associated lymphoproliferative disorders and poor survival of patients developing this complication highlight the priority in finding a prompt curative treatment for ADA-SCID.

## Introduction

The development of immunodeficiency-associated lymphoproliferative disorders (IALDs), especially related to Epstein–Barr virus (EBV), is a life-threatening phenomenon. The incidence of IALD in primary immune deficiencies due to various gene defects ranges from 0.7 to 18% of patients (1). Adenosine deaminase-deficient severe combined immunodeficiency disease (ADA-SCID) is an inherited defect that results in the accumulation of enzyme substrates, such as adenosine, 2′-deoxyadenosine and deoxyribonucleotides (dAXP) leading to severe lymphopenia with absence of cellular and humoral immunity and recurrent, severe infections. High adenosine levels block the differentiation of thymocytes, induce thymic hypoplasia, and lead to apoptosis resulting in T, B, and NK depletion. Delayed and late onset forms have a wide spectrum of manifestations mostly involving immunological and autoimmune system (i.e., hemolytic anemia and immune thrombocytopenia) as the most severe manifestation (2). Since ADA gene is ubiquitously expressed, non-immunological alterations have been described frequently in ADA-deficient patients involving liver, kidney, bone, and central nervous system (3–6).

## Background

Eight cases of lymphoma have been reported to date in ADA-SCID patients, and half of them are EBV associated (7–13).

Herein, we present the first case of a fatal EBV-associated plasmablastic lymphoma, which developed in an 18-year-old ADA-SCID girl, after enzyme replacement therapy (ERT) and multiple infusions of genetically modified T cells. The patient was diagnosed with ADA deficiency at 3 months of age due to pneumonia and failure to thrive (14). PEG-ADA was started with partial benefit on immune functions and clinical conditions but the lymphocyte count remained low (450–650/μL with 200/μL T cells). No matched bone marrow (BM) donor was available. Between 1 and 5 years of age, she was treated with repeated infusions of autologous peripheral blood (PB) T lymphocytes transduced *ex vivo* with a gammaretroviral vector encoding ADA (14) (NCT00599781, GIADAl). The patient also received a small amount of autologous BM cells transduced with a similar gammaretroviral vector (differing only at a restriction enzyme site) without any conditioning, after which there was no evidence of engraftment. PEG-ADA was reduced until complete discontinuation to favor the expansion of gene corrected cells (14). Despite the improvement of T cell count and functions with evidence of antibody responses to neoantigens, ERT was restarted at a low dose due to the suboptimal systemic detoxification, maintained at 11.2–12.5 U/kg/week i.m. and continued for further 16 years. The patient experienced mainly self-limited upper respiratory tract infections and autoimmune hemolytic anemia at the age of 6 years, which was treated with high-dose methylprednisolone (15 mg/kg i.v.) followed by prednisone 2 mg/kg/die p.o. which was slowly tapered and eventually stopped 1 year after onset. A mild pulmonary restrictive syndrome with bronchiectasis and arterial hypertension were controlled with specific therapies. At the age of 17, lymphocyte and T cell counts were 800 and 600/μL, respectively. Proliferative responses to mitogens were normal, while responses to antigens (candida and alloantigens) were reduced. The TCR Vbeta repertoire by flow cytometry was polyclonal with two Vbeta families reduced. dAXP toxic metabolites were 6 nmol/mL (0.46%) on PB (normal range: <1%) (10) EBV serology and search for EBV sequence (EBV PCR) were negative.

**Figure 1:**
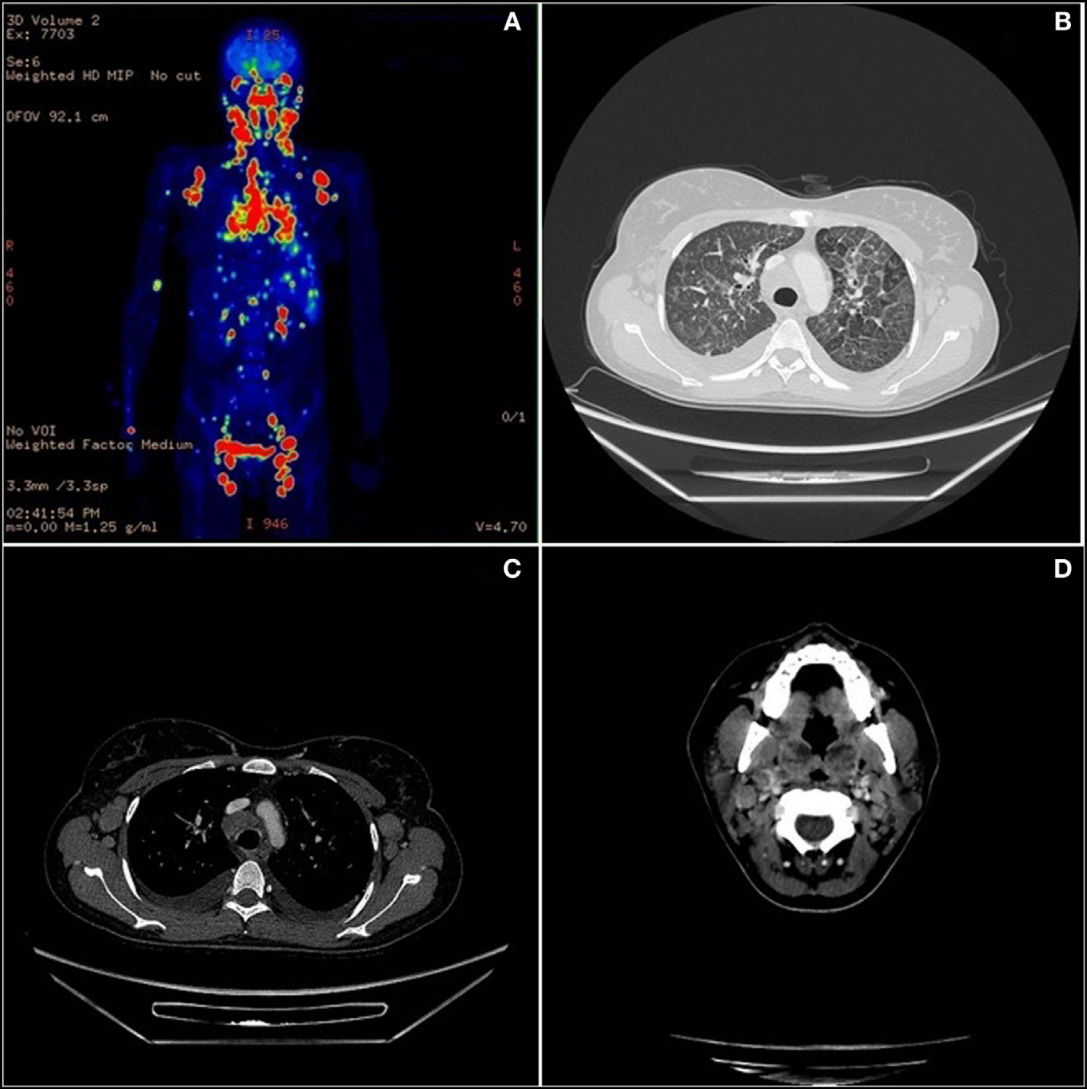
Total body positron emission tomography (PET)–computed tomography (CT). **(A)** Diffuse lymph nodes involvement in PET. **(B)** Lymphoma lung interstitial reticular thickening in CT scan. **(C)** Axillary and mediastinum enlargement lymph nodes in CT scan. **(D)** Neoplastic involvement of neck nodes and palatine tonsils in CT scan.

**Figure 2:**
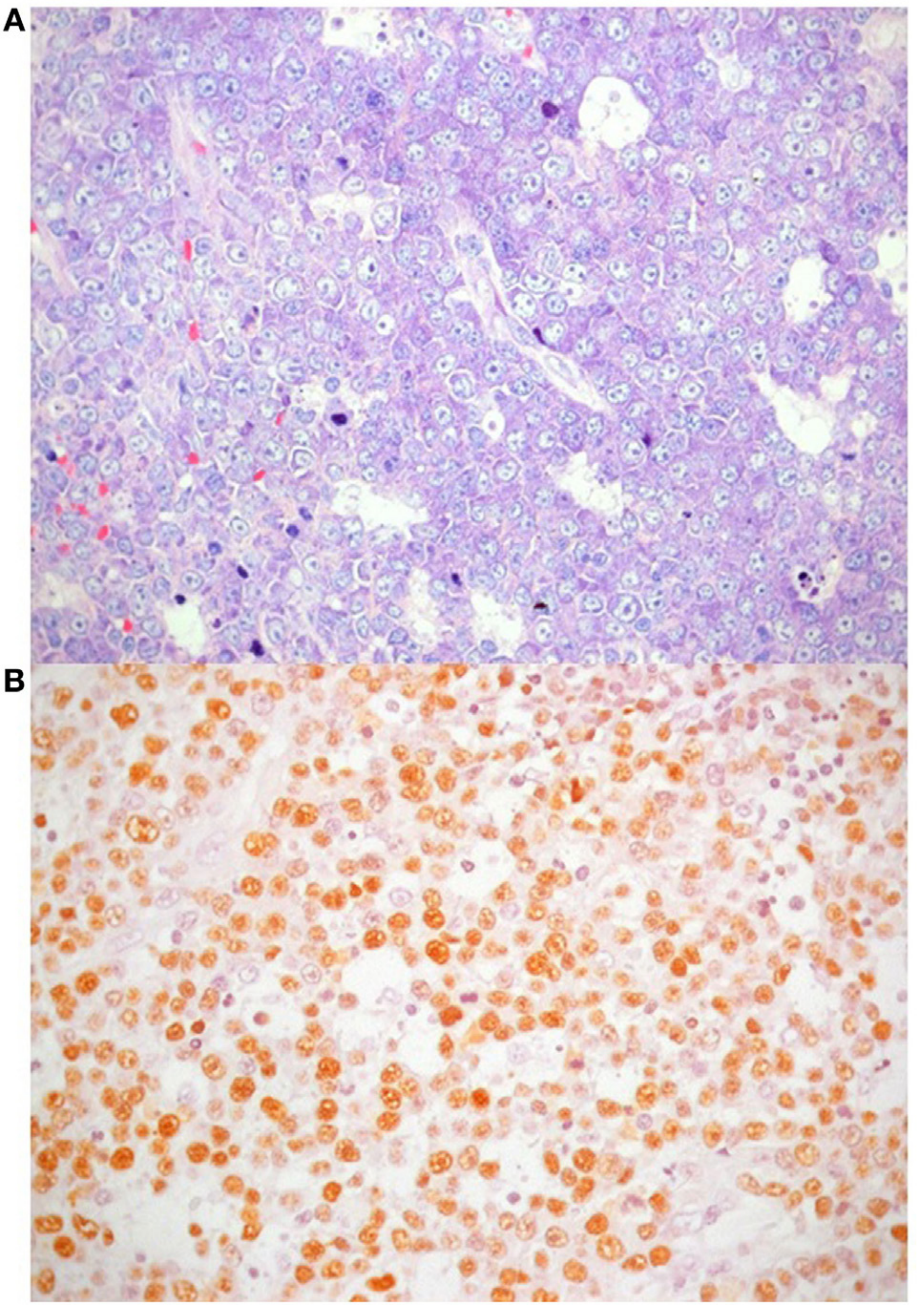
Plasmablastic lymphoma accompanied by areas of necrosis. **(A)** Giemsa stain highlights nuclear features of neoplastic cells, with particular reference to central, single prominent nucleolus (40×); **(B)** *in situ* hybridization for Epstein–Barr virus shows positive nuclear signal in neoplastic lymphocytes.

One year later the patient was hospitalized in a local hospital due to persistent fever, multiple lymphadenopathies and bilateral periorbital edema with intense conjunctival hyperemia, unresponsive to i.v. antibiotics. At admission to our Unit, total body positron emission tomography–computed tomography showed diffuse lymphadenopathies (cervical, spinal-accessorial, supraclavicular, axillary, retropectoral, tonsillar, upper mediastinal, and right paratracheal) and splenomegaly (Figure 1). Interstitial lung involvement accompanied by pleural effusion resulted in respiratory impairment, requiring oxygen supplementation. Brain MRI detected severe edema of periocular tissue with exophthalmos and lymphoid hyperplasia. Blood tests revealed high plasma EBV DNA load (10 million copies/mL) and elevated anti-VCA EBV IgG with absence of anti-VCA IgM, anti-EBV early antigen and anti-EBV nuclear antigen antibodies. An Ig kappa monoclonal component (10 g/L) was present. Cervical lymphadenectomy and BM examination were performed, and methylprednisolone (1 mg/kg/daily) and anti-CD20 (rituximab 375 mg/m^2^ in two doses) were started before pathological diagnosis was confirmed. Histopathological examination showed an EBV-related plasmablastic B-cell lymphoma (Figure 2). Lymphoma cells displayed a high proliferation index (>90%), were monoclonal for kappa light chain and expressed plasma cell-associated markers such as CD138, CD30, and MUM-1. In addition, these elements were positive for PAX-5 and BCL2 as well as partially positive (40% of cells) for CD20. The EBV marker LMP-1 was present. On the other hand, neoplastic elements scored negative for CD10, CD3, CD4, CD8, BCL6, and CD56. HHV8 was also negative. A minor component of mature-looking plasma cells sharing the same light chain restriction with large neoplastic cells was present at the edge of the lesion, as reported (15). BM evaluation showed reactive dyshematopoiesis with slight increase of plasma cells, which were monoclonal for kappa light chain.

The disease was classified as stage IV B E_s_. Therapy consisted of continued rituximab weekly infusions and a cycle of APO (adriamycin, vincristine, and prednisone) with no response. Accordingly, cyclophosphamide (300 mg/m^2^ BID for 3 days) and dexamethasone were started. Twelve days after the initiation of chemotherapy, the patient died for a hemorrhagic alveolitis eventually causing worsening respiratory failure, refractory to continuous positive airway pressure and biphasic positive airway pressure mechanical ventilation.

To investigate the potential relationship with PB gene therapy, T cells and B cells were purified from BM, PB, and lymph node, and vector transduction measured by quantitative PCR (14). T cells were highly positive for the vector (BM: >100%; PB: >100%; lymph node: 96.1%) in line with previous follow-up analyses. By contrast, B cells showed low vector signal (BM: 0.43%; PB: 3.1%; lymph node: 6.1%), a level compatible with contamination from T cells, as sample purity was <90% after purification. These data rule out the possibility that the lymphoproliferative event originated from the infused transduced cells.

## Discussion

The most frequently reported histotype in primary immune deficiencies is diffuse large B-cell lymphoma, which mostly occurs in extra nodal sites (16). Nine cases of lymphoma have been described in ADA-SCID patients, including the present case (Table 1). Of these, five were related to EBV infection (7, 10, 11) with two arising in the lung (10) and the brain (11), respectively, while another was described as a Hodgkin lymphoma (10). An EBV-negative Burkitt’s lymphoma has also been described (12). Overall, six of nine patients developed a malignant disease more than 3 years after the start of PEG-ADA (range 3–17 years) (Table 1). In three patients, alternative treatment was attempted before lymphoma onset, including haploidentical hematopoietic stem cell transplantation (HSCT) (*n* = 2) and PB T-cell gene therapy. Unfortunately, all patients died despite rescue attempts with several lines of treatments, with the exception of a Burkitt’s hip lymphoma that persisted in complete remission after 20 months from the onset of the malignant disease (12). According to data available in literature seven of nine patients received chemotherapy to cure lymphoma, mostly based on high dose of steroids (prednisone and dexamethasone), anti-mitotic vinca alkaloids (i.e., vincristine), anthracyclines (i.e., doxorubicin), alkylating agents (i.e., cyclophosphamide). Three out of ten patients underwent HSCT after lymphoma, a patient two consecutive haplo-HSCT, and the last two from cord blood and matched unrelated donor, respectively. Unfortunately, all of them (3/3) had a fatal exitus.

**Table 1:** Clinical, genetics, and biological characteristics of ADA-SCID patients who developed lymphoma (review of the published cases).

Reference	ADA mutation	PEG-ADA	HSCT for ADA-SCID	Lymphoma type	Organs involved	EBV related	Age at onset	Treatments for lymphoma	Outcome
Kapoor (7)	nk	No	Second haplo T depleted, no conditioning, poor engraftment (only T cells), and donor EBV positive	B cell of host origin, 85% k chain—50% delta chain—90% μ chain	Liver, spleen, CNS, gut, lung, and BM	Yes	1 year after II HSCT	nk	Dead 1 week after lymphoma onset
Ratech et al. (8)	nk	Yes	No	Diffuse large cell immunoblastic plasmacytoid type NHL (MALT). Monoclonal IgA lambda	Gut, spleen, nodes, liver, lung, kidney, meninges, and adrenal gland	nk	nk	nk	Died at 4 years
Hirschhorn et al. (9)	E217K; exon 1–5 deletion	No	Haplo-HSCT	nk	nk	nk	1.5 years after HSCT	2 HSCT (haplo)	Dead
Hershfield (10)	nk^a^	Yes for 13 years	No	Hodgkin lymphoma	nk	Yes	13 years after starting PEG-ADA	HSCT (CB)	Died at 16 years
Hershfield (10)	nk	Yes for 10 years	No	nk	Pulmonary nodule	Yes	8 years after starting PEG-ADA	Rituximab; HSCT (MUD) at 10 years	Died of viral disease 1 month after HSCT
Kaufman et al. (11)	A83D; Exon5 splice donor site c.573 + 1G>A	Yes for 10 years	No	B large cell type. Immunoblastic and plasmacytoid features. BCL6 neg. Deletion in 1p36, 19q13, and 10q23	CNS	Yes	10 years	DXM + phenytoin; COP + APO + 6-MP; MTX	Dead
Husain et al. (12)	Q3X (h)	Yes for 14 years	No	Burkitt’s lymphoma. CD20 and CD79a positive Aberrant co-expression T marker CD43	Right iliac-ischial bone	No	15 years	Chemotherapy not specified	Complete remission 20 months after diagnosis
Genel (13)	W272X (h)	Yes for 3 years	No	Diffuse large B cell lymphoma NHL	Multiple pulmonary nodules and splenic mass	nk	3 years	Chemotherapy (BFM 2004 protocol)	Dead
Current patient	462delG (h)	Yes for 17 years	No (infusion of gene corrected peripheral blood lymphocytes)	Plasmablastic B-cell lymphoma with plasmocytoid features. CD138, MUM-1, kappa light chain monoclonal, CD20 partially positive	Nodes, lungs, periocular, spleen, and BM	Yes	18 years	Rituximab; APO; and cyclophosphamide + dexamethasone	Dead

*HSCT, hematopoietic stem cell transplantation; EBV, Epstein–Barr virus; nk, not known; NHL, non-Hodgkin lymphoma; h, homozygous; Haplo, haploidentical; CB, cord blood; MUD, matched unrelated donor; CNS, central nervous system; BM, bone marrow; COP, cyclophosphamide, vincristine, and prednisone; APO, doxorubicin, vincristine, and prednisone; 6-MP, 6-mercaptopurine; ADA-SCID, adenosine deaminase-deficient severe combined immunodeficiency disease*.

*^a^This patient is the brother of the one described in Hirschhorn et al. (9)*.

To the best of our knowledge, this is the first report of a plasmablastic lymphoma in an ADA-SCID patient. Lymphoma in patients with primary immune deficiency is frequently EBV associated (16). EBV infection indeed seems to play an important role on lymphocyte proliferation thorough suppression of T-cell surveillance and modulation on apoptosis and cytokine balance in B-cells (8, 17).

In particular, low T-cell count and/or abnormal T-cell function seem to impact on lymphoproliferative disorder prognosis more than histology or gene rearrangements (16). Moreover, dysregulated secretion of IL-6 has a pivotal role in lymphoproliferative disorders in an autocrine and paracrine cytokine stimulated manner, indeed, it contributes to the development of the auto-maintenance and neoplastic growth of EBV-immortalized B cells (17).

The pathogenesis of lymphomas in ADA-SCID patients remains still unclear. The lack of ADA would be expected to decrease the oncogenic risk since ADA inhibitors have been used for the treatment of lymphomas (18) and the accumulation of adenosine and purine metabolites in ADA-deficient patients leads to increased apoptosis in T and B cells (19). Since all patients described here were either under PEG-ADA treatment or received HSCT, one can speculate that in these patients adenosine metabolites were adequately detoxified. It would be also interesting to investigate whether lymphoma cells spontaneously reverted the ADA mutation, as observed in selected cases of ADA-SCID patients (20). At present, the most likely explanation remains a lack of appropriate immune surveillance similarly to other primary immune deficiencies.

The B-cell clonality identified in our patient by PCR, both on lymph node and on BM, has been associated with poor prognosis, confirming the negative predictive role of this feature previously suggested in a small cohort of patients with lymphoproliferative disorders (16).

Our patient continued long-term ERT since an HLA-identical donor was not available and data of overall survival after matched unrelated or alternative donors (i.e., haploidentical) were not encouraging at that time (66 and 43%, respectively) (21). ERT often leads to a transient improvement of the immune function and clinical parameters, including growth (22, 23), but a gradual immunological decline is actually observed within a few years of treatment (23–26). Our patient also received multiple infusions of genetically corrected T cells and presented a T-cell compartment mainly represented by transduced cells. It is likely that, despite being polyclonal, the T-cell repertoire of the transduced T cells, which were *ex vivo* cultured, was not sufficiently broad to survey and control the B cell transformation induced by the EBV infection. It is also possible that defective functions of other untransduced cell types, such as NK cells, could have contributed to the impaired immune response to EBV. It will be important to continue the strict monitoring of ADA-SCID patients on long-term ERT, including those who received infusions of gene corrected T cells (27–29).

On the other hand, retroviral vector GT with autologous CD34+ cells transduced with *ADA* has emerged as a safe and efficacious therapeutic strategy for patients lacking HLA-identical sibling donor (26, 30–33). Noteworthy, in comparison with clinical trials conducted on other primary immunodeficiencies (SCIDX1, CGD, and WAS), no lymphoproliferative disorders or aberrant clonal expansion have been observed in more than 40 patients treated worldwide with an available follow-up of more than one decade in some of them (34).

## Concluding Remarks

These observations underline the importance of defining a potential lifelong effective treatment for these patients as early as possible. Attending physicians must be aware that the delay of a definitive treatment could expose these fragile patients to multiple severe complications, including aggressive lymphoproliferative disorders.

## Ethics Statement

The study was performed in accordance with the Declaration of Helsinki. The patient was enrolled in a clinical trial approved by the San Raffaele Ethical Committee (NCT00599781). Written informed consent was obtained from parents for treatment, biological sample collection, and data publication.

## Author Contributions

M.M., AndreaA., M.P., R.P, F.B., F.G., F.F., M.F., I.B., F.D., R.N., M.C., M.G.R., C.D., J.P., F.C., M.P.C, AlessandroA. participated to clinical care of the patient and related clinical data. M.M. and AlessandroA. designed the study, analyzed results and wrote the paper.

## Conflict of Interest Statement

AlessandroA is the Principal investigator of gene therapy studies sponsored by GlaxoSmithKline (GSK), which holds the licence for the medicinal product based on autologous CD34+ cell gene therapy for ADA-SCID. All other authors declare that the research was conducted in the absence of any commercial or financial relationships that could be construed as a potential conflict of interest.
